# Modeling respiratory tract diseases for clinical translation employing conditionally reprogrammed cells

**DOI:** 10.1016/j.cellin.2024.100201

**Published:** 2024-09-18

**Authors:** Danyal Daneshdoust, Kai He, Qi-En Wang, Jenny Li, Xuefeng Liu

**Affiliations:** a Comprehensive Cancer Center, The Ohio State University, Columbus, OH, USA; b Division of Medical Oncology, Department of Medicine, Wexner Medical Center, The Ohio State University, Columbus, OH, USA; c Department of Radiation Oncology, Wexner Medical Center, The Ohio State University, Columbus, OH, USA; d Department of Pathology, Wexner Medical Center, Ohio State University, Columbus, OH, USA; e Departments of Pathology, Urology, and Radiation Oncology, Wexner Medical Center, Ohio State University, Columbus, OH, USA

**Keywords:** Conditional reprogramming, Preclinical models, Translational medicine, Respiratory diseases, Lung cancer, Asthma, Cystic fibrosis, Respiratory viral infections, Covid-19, Respiratory papillomatosis

## Abstract

Preclinical models serve as indispensable tools in translational medicine. Specifically, patient-derived models such as patient-derived xenografts (PDX), induced pluripotent stem cells (iPSC), organoids, and recently developed technique of conditional reprogramming (CR) have been employed to reflect the host characteristics of diseases. CR technology involves co-culturing epithelial cells with irradiated Swiss-3T3-J2 mouse fibroblasts (feeder cells) in the presence of a Rho kinase (ROCK) inhibitor, Y-27632. CR technique facilitates the rapid conversion of both normal and malignant cells into a “reprogrammed stem-like” state, marked by robust in vitro proliferation. This is achieved without reliance on exogenous gene expression or viral transfection, while maintaining the genetic profile of the parental cells. So far, CR technology has been used to study biology of diseases, targeted therapies (precision medicine), regenerative medicine, and noninvasive diagnosis and surveillance. Respiratory diseases, ranking as the third leading cause of global mortality, pose a significant burden to healthcare systems worldwide. Given the substantial mortality and morbidity rates of respiratory diseases, efficient and rapid preclinical models are imperative to accurately recapitulate the diverse spectrum of respiratory conditions. In this article, we discuss the applications and future potential of CR technology in modeling various respiratory tract diseases, including lung cancer, respiratory viral infections (such as influenza and Covid-19 and etc.), asthma, cystic fibrosis, respiratory papillomatosis, and upper aerodigestive track tumors. Furthermore, we discuss the potential utility of CR in personalized medicine, regenerative medicine, and clinical translation.

## 1 Introduction

Respiratory tract diseases consist of a broad range of conditions that impact the lungs, upper and lower airways. These conditions include tumors, asthma, chronic obstructive pulmonary disease (COPD), cystic fibrosis (CF), idiopathic pulmonary fibrosis, respiratory infections, etc. Respiratory diseases, being the third most common cause of death worldwide, pose a significant burden to healthcare systems and societies (Global burden of 369 diseases, 2020). The five respiratory diseases that cause the majority of the world's health problems include lung cancer, asthma, COPD, tuberculosis, and acute respiratory infections (Chan et al., 2023). With over 775 million confirmed cases of COVID-19 including more than 7 million deaths across the world, had been reported to the World Health Organization (WHO), the severe acute respiratory syndrome coronavirus-2 (SARS-CoV-2) remains a major global health concern (Guan et al., 2020; Msemburi et al., 2023; WHO. 2024, 2024). Given the substantial mortality and morbidity rates of respiratory diseases, efficient and rapid preclinical models are imperative to accurately recapitulate the diverse spectrum of respiratory conditions. These models are essential for investigating developmental processes, physiology, and physiopathology of respiratory tract disorders, as well as for discovering the most efficient drug therapeutics.

Respiratory diseases have historically been studied using established cell lines cultured in conventional two-dimensional (2D) models. While these 2D models have greatly contributed to progress in respiratory system research, they are not without limitations. Only 1%–10% of cell lines can be successfully transformed into immortalized or cancer cell lines, primarily due to the short lifespan of most primary cells, particularly normal cells (Giard et al., 1973). Furthermore, findings derived from traditional 2D cultures may not faithfully resemble the host response, as the genes of established cell lines undergo substantial alterations over prolonged periods of culturing (Gillet et al., 2013).

The limitations of cell lines may be overcome by using animal models, particularly the more recently developed primary patient-derived xenografts (PDX) and engineered mouse models, which more closely resemble human disease and treatment responses (Walsh et al., 2017). Animal models are frequently employed in laboratory research and have contributed significantly to our knowledge of respiratory disease biology (Ito et al., 2019). Furthermore, animal models have been essential in preclinical research involving various respiratory diseases such as cystic fibrosis, lung cancers, and viral airway infections (Guilbault et al., 2007; Huo et al., 2020; Meraz et al., 2019; Sefik et al., 2022). Nevertheless, the translation of experimental results from animal models to clinical settings can be time consuming due to high costs, low throughput, and technical difficulties. Moreover, species-specific variations lead to inaccurate recapitulation of biological and therapeutic responses. To address these issues, translational medicine models that are rapid, efficient, and robust and can mimic the original disorder (phenotypic and genotypic) must be developed.

Recent advancements in biotechnology have imposed significant changes in the development of preclinical disease models. One particularly promising approach is the use of patient-derived models (PDM), which maintain genetic characteristics consistent with the parental tissues. Different PDM types, including PDX, iPSC, organoids, and conditionally reprogrammed cell (CRC), have been used extensively in preclinical research, since they recapitulate more accurately the complexity of human tissues (Borodovsky et al., 2017; Hui et al., 2018; Okada et al., 2019; Rowe et al., 2019). Depending on the setting and techniques used, PDM can be employed in preclinical research in various ways (Bukowy-Bieryłło, 2021). In this review, an extensive discussion of the CR technology is provided along with a comparison of these models (Table 1). This article provides a comprehensive overview of the current state and potential applications of the CR method in respiratory system disorders.

**Table 1:** | Comparisons between models: Conventional cell lines (2D culture), organoids, iPSC, PDX, and CRC.

Models	Advantages	Shortcomings
Conventional cell lines (2D)	1. Low-cost technique 2. Simple genetic manipulation	1. Tumor heterogeneity loss 2. Absence of microenvironment
Organoids	1. 3D culturing 2. Able to produce from both normal and malignant 3. Retain tumor heterogeneity	1. Dependent on stem cells 2. Excessive proliferation of benign cells
iPSC	1. Pluripotent differentiation 2. Can combine with gene editing and 3D organoids	1. Slow and inefficient procedure 2. Difficult to reprogram cancer cells 3. Safety issues
PDX	1. In vivo model 2. Direct engraftment from human tumor 3. Resemble the natural environment of the tumor 4. Preserve histological, genomic, and transcriptomic features of tissue of origin	1. Expensive technique 2. Resource and time consuming 3. Unsuitable for normal tissue expansion 4. Unsuitable for high-throughput therapeutics screening 5. Depend on interacting with a mouse's environment
CRC	1. A wide range of specimen sources 2. Both normal and malignant cell culturing 3. High-throughput drug screening 4. Retain original karyotype and tumor heterogeneity 5. Gene profiling analyses 6. Rapid expansion	1. Contamination with feeder cells 2. Overgrowth of benign cells 3. Lack of stromal components (matrix elements, vascular immune cells)

## 2 CR technology

**Fig. 1:**
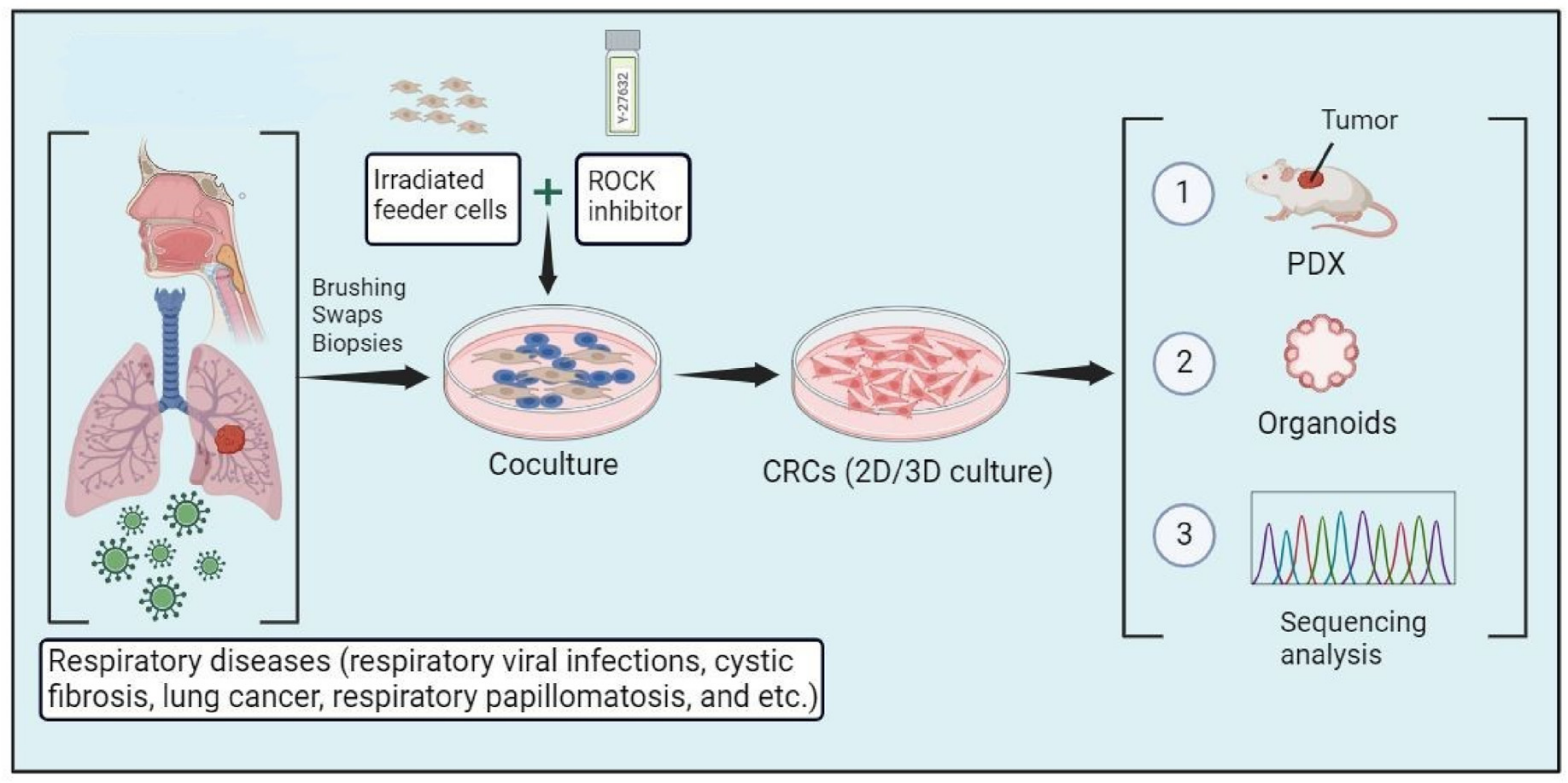
**CR technology has significant applications in respiratory tract disorders. The CR method enables the rapid generation of cultures from both normal and malignant tissue obtained through brushings, swabs, and biopsies.** The figure was created using BioRender.

We have recently developed a novel primary cell culture method known as conditional reprogramming. This innovative technique allows the rapid, efficient, and continuous expansion of both normal and cancerous cells derived from patients (Liu et al., 2017). CR technology is co-culturing epithelial cells with irradiated Swiss-3T3-J2 mouse fibroblasts (feeder cells) in the presence of a ROCK inhibitor, Y-27632 (Fig. 1). The CR technique rapidly induces normal and tumor cells into a “reprogrammed stem-like” state, characterized by high proliferation rates while maintaining their original karyotypes. Notably, eliminating these conditions reinstates the cells' ability for differentiation, and the phenotype becomes entirely reversible (Liu et al., 2012; Suprynowicz et al., 2012). The CR technique demonstrates its effectiveness in generating large quantities of primary cells from various tissue sources, including fine-needle aspiration (FNA), core biopsies, surgical specimens, brushings, swabs, urine samples, and patient-derived xenograft tissues (Palechor-Ceron et al., 2019). In brief, tissue samples derived from patients undergo initial histological evaluation by a pathologist to assess their composition. Subsequently, the samples undergo mechanical fragmentation followed by enzymatic digestion to disperse them into single cells, which are then plated in a medium supplemented with irradiated Swiss-3T3-J2 mouse fibroblasts (feeder cells) and a ROCK inhibitor. Epithelial colonies readily become discernible within two days and usually exhibit rapid proliferation, achieving confluence in approximately 5 days. CR technology enables the establishment and culture of 2 million epithelial cells in only 5–6 days and continues to passage for 100 population doubling over ≥110 days (Liu et al., 2014, 2017; Zhong et al., 2020).

Cells generated using this method are referred to as Conditionally Reprogrammed Cells (CRCs). CR technology is characterized by several notable features, including genetic stability, high success rates within a single model system, exponential expansion capabilities, and straightforward implementation (Liu et al., 2020a; Wu et al., 2020a). CRCs have the capability to reflect the genetic and histological properties of the parental tissue, thereby enabling them to sustain a highly proliferative state (Alamri et al., 2016). An important aspect is the versatility of these cultures, which can be employed for establishing xenografts (Suprynowicz et al., 2012), patient-derived xenograft (PDX) cell lines (Mondal et al., 2019), cell cultures derived from PDXs, and organoid cultures. Conditional reprogramming ensures the preservation of cell lineage commitment and the cellular heterogeneity inherent in a biopsy specimen (Liu et al., 2012; Saeed et al., 2017).

The aforementioned characteristics of CR qualify this model as an outstanding in vitro model compared to other models such as organoids and patient-derived xenografts (Palechor-Ceron et al., 2019). Modeling diseases, precision medicine, drug discovery, regenerative medicine, and noninvasive diagnosis are some of the applications of CR technique (Jung et al., 2022; Liu et al., 2020a; Stover et al., 2022). Clinical and translational research applications of CR technology have been investigated in various diseases such as, lung cancer (Gao et al., 2017), cystic fibrosis (Martinovich et al., 2017), upper respiratory viral infections (Liu, Wu, & Rong, 2020), Liu, Wu, & Rong, 2020reast cancer (Daneshdoust et al., 2023a), prostate cancer (Timofeeva et al., 2017), bladder cancer (Daneshdoust et al., 2023b), and digestive system disorders (Zhao et al., 2021). Furthermore, CR technology has shown its potential in rare conditions such as respiratory papillomatosis (Yuan et al., 2012) and salivary cancer (Alamri et al., 2018). Furthermore, the utility of this system extends beyond humans; it can be applied to various mammalian species, including mice, rats, ferrets, horses, cows, and dogs (Daneshdoust et al., 2023b; Saffari et al., 2019).

## 3 Potential applications in modelling respiratory tract diseases

### 3.1 Respiratory viral infections

Respiratory viral infections are a major cause of death in the current century (Kang et al., 2023). Respiratory viruses include influenza virus, parainfluenza virus, rhinovirus, respiratory syncytial virus (RSV), adenovirus, and coronavirus. Except for adenoviruses, which have double-stranded DNA genomes, all these viruses have single-stranded RNA genomes. Immunocompromised cancer patients face a higher mortality rate following respiratory viral infections (Greenberg, 2002). Moreover, these infections are closely associated with exacerbations of asthma and COPD (Bhatt et al., 2023; Gern, 2008). In December 2019, SARS-CoV-2, new variant of coronavirus, emerged and caused a pandemic of COVID-19 (Soltani-Zangbar et al., 2023). The COVID-19 pandemic has highlighted the need for novel preclinical airway models to study the pathogenesis of respiratory viruses and develop vaccines and therapies.

**Fig. 2:**
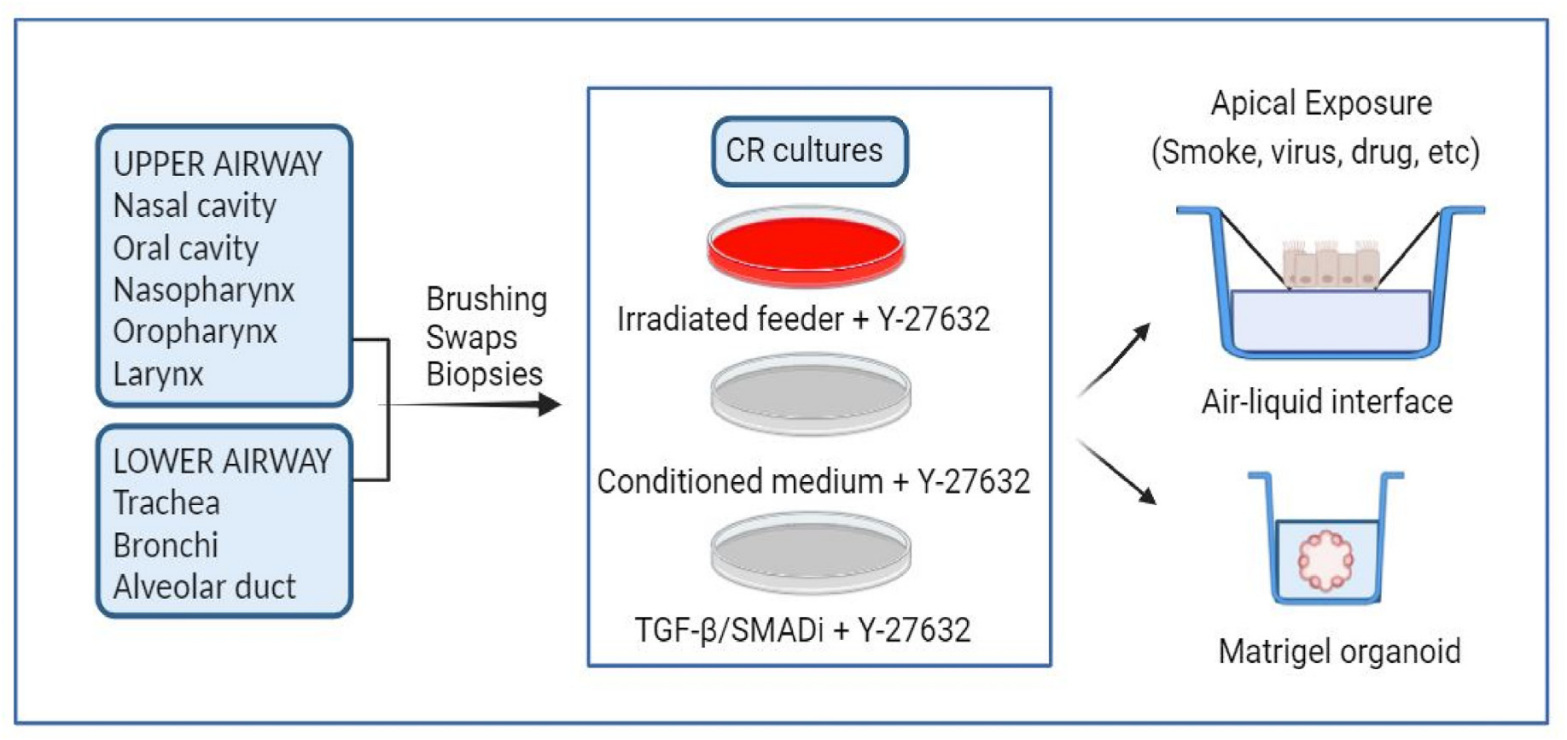
**|Workflow of human normal airway epithelial cells CRC culture under in vitro apical (ALI) and closed organoids (3D) cultures.** The figure was created using BioRender.

CR technology has been utilized independently or with air-liquid interface (ALI) cultures to create preclinical models that evaluate host-viral interactions (Liu, Wu, & Rong, 2020). The ALI method allows cells to differentiate morphologically and functionally to reflect their natural environment by exposing their apical surfaces to air while their basal surfaces remain submerged in culture medium (Fig. 2) (Rani et al., 2023; Tata et al., 2013). This method accurately mimics the in vivo airway, making ALI cultures of airway CRCs ideal for in vitro infection studies. Tran et al. collected samples from nasal turbinate brushing, cultured under CR conditions, and differentiated them in ALI culture (Tran et al., 2022). The differentiated cells were subsequently inoculated with various clinical isolates of SARS-CoV-2. Consistent and reproducible infection of the ALI nasal epithelium, derived from different donors, was observed. Their findings indicated that ALI nasal epithelium serves as a reliable model for SARS-CoV-2 infection and can accurately predict the pathogenicity of mutant SARS-CoV-2 variants. This study demonstrates that integrating CR with ALI creates a robust technique to mimic human SARS-CoV-2 infection in nasal epithelium, serving as a valuable preclinical tool without needing invasive human tissue samples (Tran et al., 2022). Roberts and colleagues expanded bronchial and nasal epithelial cells collected via brushing under CR conditions and seeded the passaged cells onto collagen-coated transwells for ALI culture (Roberts et al., 2018). Their findings revealed that viral infection in both cell types led to an increase in the expression of interferon gamma-induced protein 10 (IP-10), although the elevation was statistically significant only in the ALI culture, particularly when combining rhinovirus infection with IL-13 treatment (Roberts et al., 2018). Robert et al.'s study demonstrates that nasal and bronchial airway epithelial cells can be used interchangeably in models of rhinovirus infection and IL-13 treatment. In another investigation, Schmidt et al. evaluated the feasibility of ALI culture using nasal epithelial CRCs as a model to study SARS-CoV-2 infection and its effects on ion and water transport (Schmidt et al., 2022). They found that ALI epithelia retained major differentiation characteristics and physiological ion and water transport properties across passages, indicating that passaged nasal epithelial CRCs are feasible for studying SARS-CoV-2 infection and epithelial transport function (Schmidt et al., 2022).

The ongoing SARS-CoV-2 pandemic, marked by the emergence of new variants with adaptive mutations, has led to the “replacement” of previous strains. Recently, Meganck et al. investigated the growth, adaption, and replicative fitness of SARS-Cov-2 variants including D614G, Alpha, Gamma, Delta, and Omicron in primary human airway epithelia generated under CR conditions and cultured in the ALI system (Meganck et al., 2024). Several notable differences were reported when analyzing the growth of SARS-CoV-2 variants across primary large airway, small airway, and nasal epithelia. In lower airway cells, the variants D614G, Alpha, and Delta showed sustained viral replication efficiency for 96 h, while Omicron reached its peak growth at 48 h. Ciliated cells were identified as the primary infection targets, as confirmed by various independent methods. Additionally, Alpha, Gamma, and Delta variants demonstrated enhanced fitness compared to D614G. Conversely, Omicron's global dissemination is likely due to antigenic variability and higher transmission (Meganck et al., 2024).

To evaluate morphology, barrier function, interferon production, and gene expression following human rhinovirus A1 infection, Veerati and colleagues compared bronchial epithelial cells derived from asthma donors with their corresponding CR cells at terminal differentiation (Veerati et al., 2022). They found that CR cells differentiated in ALI cultures recapitulated their parental cells' features, except for the superficial elongation of the cell cytoskeleton to basal cells, and showed no difference in barrier function compared to their parent cell cultures (Veerati et al., 2022). Future research should continue to explore the potential of CRCs in studying lung viral infections, leveraging these preclinical models to uncover novel therapeutic targets and improve our understanding of respiratory virus pathogenesis.

### 3.2 Cystic fibrosis

The inherited disorder known as CF, caused by different mutations in the gene encoding CF transmembrane conductance regulator (CFTR), affects several organ systems, including the respiratory, reproductive, and digestive systems in children and young adults. Mutations in the electrolyte transport across the membranes of epithelial cells are considered to be the cause of the enhanced viscosity of mucous secretions (Cutting et al., 1992). In the United States, people with CF experience a heavy financial burden associated with unmet medical needs. The economic burden of CF on individuals and society is substantial (Seyoum et al., 2023). CF human bronchial epithelial cells play a pivotal role in developing novel CFTR modulator therapeutics. Gentzsch et al. employed the CR method to expand F508 del homozygous CF human bronchial epithelial cells (Gentzsch et al., 2017). Human bronchial epithelial cells grown under CRC conditions grew exponentially to approximately 25 population doublings within 10 passages, while cells grown under conventional conditions underwent senescence within ∼10 population doublings. Confluent ALI cultures were formed when highly expanded F508 del homozygous cells were placed on porous supports and exposed to differentiation conditions. There was a significant CFTR rescue after the cultures were treated with VX-809. They also observed morphological and functional changes resulting from extended population doublings. Consequently, CRC-expanded human bronchial epithelial cells might differ in certain aspects of complex cell biological mechanisms and may not accurately represent less extensively expanded cells in all study types (Gentzsch et al., 2017). Martinovich et al. carried out a study to investigate if airway epithelial cells obtained from patients with CF and asthma, as well as from healthy children under CRC condition could preserve their lineage during expansion and if this was affected by the underlying status of the disease (Martinovich et al., 2017). They demonstrated that airway epithelial cells with conditional reprogramming could be generated from both healthy and diseased phenotypes. Airway epithelial cells that have undergone conditional reprogramming can be grown, cryopreserved, and preserve at least five passages while maintaining their phenotypic characteristics. CR airway epithelial cell cultures had population doublings that were substantially higher than those of standard cultures while still retaining their lineage characteristics. All phenotypes of CR airway epithelial cells were also able to fully differentiate at ALI and retain disease-specific characteristics. According to their research, children's CR airway epithelial cells retain their lineage, phenotypic, and most importantly, disease-specific functional characteristics over a specified passage range (Martinovich et al., 2017). This study demonstrates that the CR method enables the rapid expansion and enhanced longevity of patient-specific airway epithelial cell cultures, providing a valuable tool for better understanding disease pathobiology. The most common pathogenic variant of CF is F508del. Currently, Trikafta, an FDA-approved medication, is accessible for these patients. Trikafta, a triple combination of Tezacaftor, Elexacaftor, and Ivacaftor, is very effective in improving respiratory function and reducing exacerbations (Bear, 2020). Consequently, this treatment is available to most CF patients. However, most genotypes that do not carry the F508del allele are not eligible to receive Trikafta or other treatments. The L1077P CFTR pathogenic variant is one of these uncommon genotypes that, when paired with another CF-causing variant, results in severe disease. Patients who have the genotype variation L1077P experience intestinal issues, chronic respiratory inflammation, recurrent respiratory infections, and pancreatic insufficiency. Additionally, they require oral supplements to aid in the absorption of nutrients and vitamins, as well as oral pancreatic supplements to assist with digestion (Bear, 2020). To characterize the L1077P CFTR variant and assess its response to the combination of Tezacaftor, Elexacaftor, and Ivacaftor, Lo Cicero et al. employed patient-derived nasal epithelial CRCs, 3D organoids, and ALI culture models of CF (Lo Cicero et al., 2023). They studied cells from two distinct patients with the homozygous L1077P/L1077P genotype and the compound heterozygous L1077P/W1282X genotype, using as comparisons the genotypes W1282X/W1282X and F508del/F508del. Protein analyses, organoid swelling induced by forskolin, and Ussing chamber assays all confirmed that the combination of Tezacaftor, Elexacaftor, and Ivacaftor was able to rescue the L1077P variant's function (Lo Cicero et al., 2023). Aforementioned study demonstrates the application of CR in precision medicine as well as in rare variants of diseases. Awatade and colleagues collected samples of nasal epithelial brushings from 5 non-CF and 9 CF individuals (Awatade et al., 2021). Subsequently, expanded under CRC condition and feeder-serum-free “dual- SMAD inhibition” (SMADi) technique. However, both CRC and SMADi cells exhibited the characteristic epithelial cobblestone phenotype, their morphologies differed during the expansion phase. Their research revealed that there were significant similarities between the population doubling times of SMADi and CRC. There was no significant difference between the two methods in terms of mucociliary differentiation markers. a significantly lower Cilia beat frequency was observed in the SMADi ^ALI^ when compared to the CRC ^ALI^. The SMADi^ALI^ exhibited a significant reduced cilia beat frequency in contrast to the CRC^ALI^ (Awatade et al., 2021).

### 3.3 Lung cancer

Lung cancer is the leading cause of cancer-related death worldwide (Leiter et al., 2023). The most important characteristic of all malignancies is intra-tumor heterogeneity, which is defined by the coexistence of genetically distinct sub-clonal populations of cells within the same tumor and determines the primary tumor's progression and response to treatment (Jamal-Hanjani et al., 2017; McGranahan et al., 2017; Prasetyanti et al., 2017; Rybinski et al., 2016). Since most tumors are clonal, traditional cell line models are unable to accurately represent this key feature. PDX models can capture intra-tumor heterogeneity; however the success rate of establishing these models are low (Cassidy et al., 2015; Rosfjord et al., 2014; Sun et al., 2016). CR technology has emerged as a promising alternative. Correa et al. collected samples from 10 primary lung cancer patients and expanded them under CR conditions. Subsequently, they tested copy number variations and performed whole exome sequencing (Correa et al., 2018). This study demonstrated CR technique's efficacy by preserving the morphological characteristics and capturing intra-tumor heterogeneity in non-small cell lung cancer (NSCLC) cells from primary tumors (Correa et al., 2018). Bordovsky et al. extended this approach to human lung PDX tumors, successfully generating CR cells that maintained genetic fidelity when transplanted into NOD/SCID/gamma mice (Borodovsky et al., 2017). By using the CR method, they created a platform to develop physiologically relevant and predictive preclinical models, thereby advancing drug discovery efforts. Crystal et al. explored the therapeutic implications, highlighting effective treatment strategies for resistant NSCLC variants using CR-derived cells. They reported that using CR cells derived from lung cancer patient resistant to EGFR and ALK tyrosine kinase inhibitors, combined ALK and MAPK kinase inhibitors was effective in an ALK-positive resistant cancer with an activating mutation in MAP2K1, and combined EGFR and FGFR inhibitors was effective in an EGFR-mutant resistant tumor with a FGFR3 mutation. In lung cancer driven by ALK, the combination of SRC and ALK inhibition functioned as well (Crystal et al., 2014). In this study, NSCLC CR cells were developed with a ∼50% success rate from advanced lung cancer samples (effusions and biopsies), initially with feeder cell cultures. CR cells were then subsequently passaged off the feeder layer for further expansion. It's noted that the major failures were due to low cancer cellularity in the specimens. In the majority (24/39) of the “failures”, the specimens contained less than 20% cancer cells. Despite challenges in establishing CR cells from low-cellularity specimens obtained via CT-guided needle biopsies, successful models retained key mutations and resistance profiles observed in patient tumors (Crystal et al., 2014; Piotrowska et al., 2015).

Tang et al. further characterized lung adenocarcinoma CR cells, revealing mechanistic insights into therapeutic responses such as Pristimerin-induced apoptosis pathways (Tang et al., 2020). Pristimerin prevented lung adenocarcinoma CR cells (LACRCs) from proliferation by inhibiting cell migration, invasion, viability, and the development of capillary structures. Tang et al.‘s research offered mechanistic data that the downregulation of EphB4/CDC42/N-WASP signaling contributed to the stimulation of Pristimerin-induced endoplasmic reticulum stress and mitochondrial-mediated intrinsic apoptosis, which in consequently resulted cellular damage in LACRCs. This study underscore CR technology's potential in modeling patient-specific cancer behaviors and responses to therapy. (Tang et al., 2020). Wu et al. used CR technology for the first time to expand cancer cells from the pleural effusion of a patient who had epidermal growth factor receptor-mutant NSCLC with primary resistance to tyrosine kinase inhibitors. Afterwards, these cancer cells were used for drug testing (cisplatin and pemetrexed) to identify the best combination regimen for the patient. It's interesting to note that the patient's response to clinical treatment and the in vitro drug response were correlated (Wu et al., 2020b). In summary, CR technology holds promise for enhancing precision medicine approaches in lung cancer research. Future directions should focus on refining methodologies to overcome specimen challenges and expanding application across diverse patient populations.

### 3.4 Upper aerodigestive track malignancies

Globally, the incidence of upper aerodigestive track malignancies is increasing (Ferlay et al., 2019). Upper aerodigestive track tumors originates from the mucosal epithelium of the oral cavity, pharynx, and larynx (Pfister et al., 2011). Druggable targets for upper aerodigestive track tumors have not been identified through analysis of the mutational landscape, and the current treatments frequently involve radiation, chemotherapy, and surgery. While aggressive chemotherapy can be effective, relapse rates may rise above 50%. Recently, immunotherapy has become a viable treatment option for upper aerodigestive track tumors; some encouraging results with immune checkpoint therapy have been documented (Haddad et al., 2018; Hill et al., 2019). Due to the heterogeneity of upper aerodigestive track tumors, there is an urgent need for reliable preclinical models, which can identify treatment strategies for patients.

Recently, Dong and colleagues investigated the feasibility of CR method in laryngeal and hypopharyngeal cancers (Dong et al., 2021). Twenty-eight laryngeal/hypopharyngeal CR tumor cells from 50 tumor tissues were successfully established using CR technique. These cells maintained tumorigenic potential upon xenotransplantation and molecular characteristics of laryngeal/hypopharyngeal squamous cell carcinomas. CR cells were able to be transformed to organoid and xenograft, and they shared comparable drug responses. The clinical drug responses in patients were consistent with in vitro drug responses (Dong et al., 2021). Li et al. employed the CR technique to establish HNSCC tumor cell cultures from 19 patients (Li et al., 2023). These tumors were established from a series of consecutively enrolled patients representing the diverse racial and ethnic population. Cell lines were derived from tumors arising in anatomical sites typical of HNSCC, including both HPV-positive, and HPV-negative tumors. The authenticity of cell lines was validated using short tandem repeat (STR) genotyping versus patient tumor tissue DNA. Out of the 19 tumors, 16 were successfully cultured. Among the 16 CR cultures, 9 originated from the oral cavity, 4 from the oropharynx, and 3 from laryngeal carcinomas. Expression of ΔNp63 and cytokeratin 5/6, indicative of squamous identity, was observed in all CR cultures. HPV presence was determined through RT-PCR of HPV16/18-specific viral oncogenes E6 and E7 in RNA extracted from tumor samples, as well as HPV DNA sequencing. Three out of 4 oropharyngeal tumors tested positive for p16 and HPV and maintained HPV in culture. CR cultures were capable of forming three-dimensional spheroids and murine flank and orthotopic tongue models (Li et al., 2023). According to Ow et al.'s study, HNSCC can be reliably established from all major aerodigestive sites using CR culture methods (Ow et al., 2024). Ow and colleagues collected tumor and blood samples from 44 head and neck squamous cell cancer patients. Then established successfully 31 (70%) CR cultures. Since at least two of the first four samples had fungal contamination, which prevented them from growing in culture, the subsequent cultures were cultivated under stricter control for the first 48 h and underwent intensive fungal treatment with nystatin and/or amphotericin. Among the 40 subsequently enrolled subjects, CR culture proved successful in 78% of cases. Both tumor type (with a notably higher success rate observed among first primary, non-recurrent tumors) and ethnicity (with a stronger association found among tumors from Hispanic patients) exhibited significant correlations. From p16-positive oropharyngeal cancers, successful CR lines were established in 66% (8 out of 12), while only 25% (1 out of 4) of p16-negative oropharyngeal cancers yielded successful cultures. Among these, three CR cultures continued to express E6 and E7 RNA as well as HPV-DNA. Following HPV genotyping, these three cultures tested positive for HPV-16. Notably, one sample harbored HPV-69, a rare but oncogenic variant that persisted in CR culture (Ow et al., 2024).

Nasopharyngeal carcinoma is an invasive cancer and prevalent in endemic areas such as Southeast Asia and Southern China (Yu et al., 2002). Primary cultures of nasopharyngeal carcinoma are known to be challenging to expand. Yu et al. sought to establish nasopharyngeal carcinoma patient-derived primary tumor cells using CR method (Yu et al., 2017a). They showed that epithelial cells could be effectively expanded from nasopharyngeal biopsies that were cancerous (46%) and normal (70%). However, the epithelial cells obtained from the cancerous biopsy represented non-malignant cells when compared to the original tumors in terms of mutation and methylation profiles. Additionally, their cell surface proteins demonstrated stem-like properties, and in an air-liquid interface culture system, they could differentiate into pseudostratified epithelium (Yu et al., 2017a). This study demonstrated the CR method could be an ideal model to cultivate non-malignant nasopharyngeal epithelial cells.

### 3.5 Respiratory papillomatosis

**Fig. 3:**
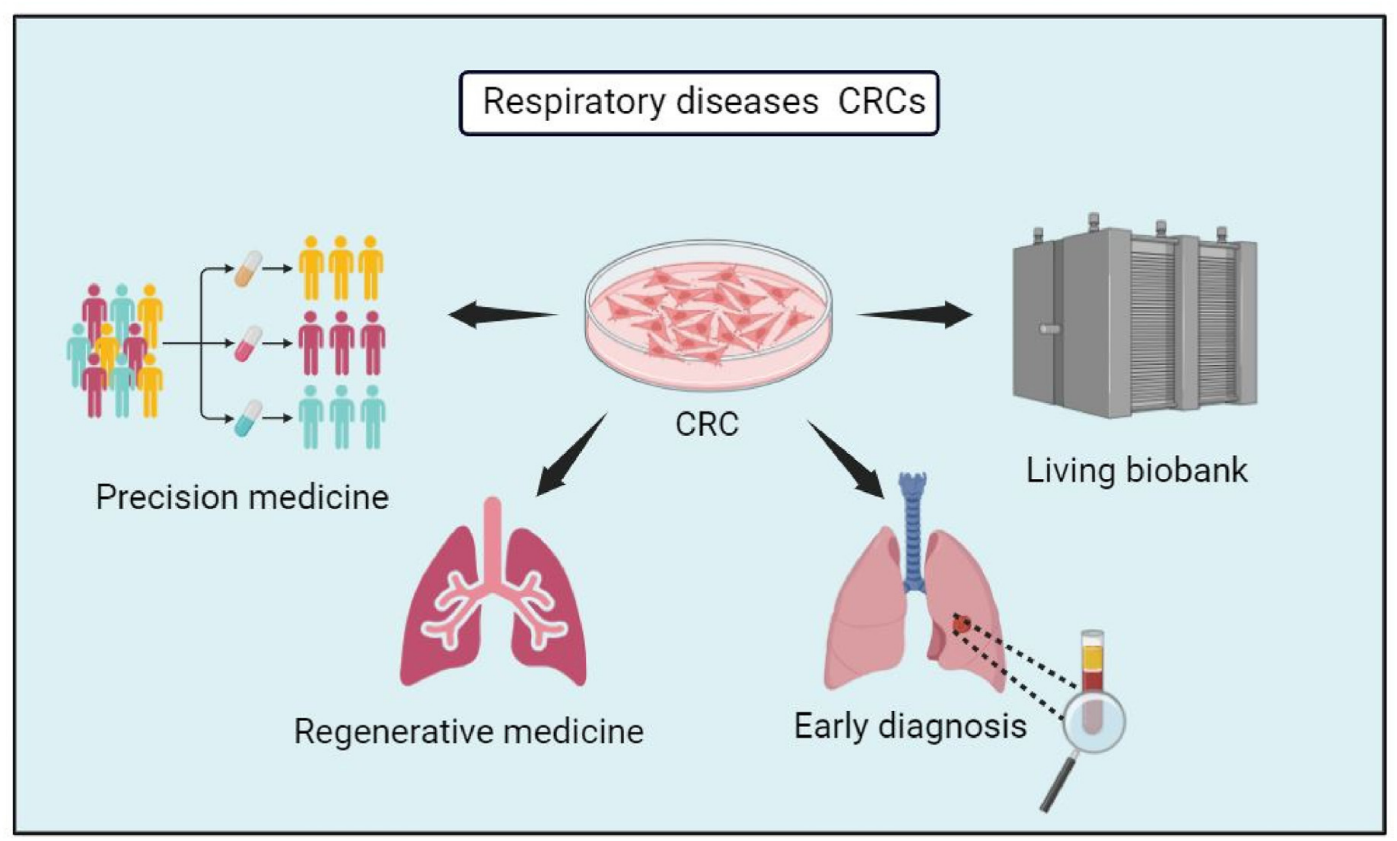
**|Applications of CRCs in respiratory tract diseases.** The figure was created using BioRender.

In 2012, The New England Journal of Medicine published an article exploring the utilization of CR technology in assessing effective treatments for recurrent respiratory papillomatosis (Yuan et al., 2012). A 24-year-old patient with recurrent respiratory papillomatosis had taken several chemotherapy agents and undergone over 350 laryngeal ablation surgeries, but all strategies had failed. The CR technique was approved for the patient's paired normal and tumor cells to be cultured for drug screening in order to control the progressive and chemoresistant disease. Consequently, the researchers found that the mutant HPV-11 genomes in the lung tumor CRCs and the laryngeal tumor CRCs were of different sizes, and Vorinostat was found to be an effective treatment. Interestingly, the tumor sizes had stabilized after three months of Vorinostat treatment. This case indicates that the CR technology holds significant promise for advancing individualized precise medicine and rare diseases (Fig. 3) (Yuan et al., 2012).

## 4 Important factors that impact CR success

### 4.1 Pathology evaluation is critical for surgical specimens that are used for CR cultures

As described in the original protocol in Nature Protocols, CR culture allows outgrowth of both normal and tumor cells from surgical specimens which is similar to organoid cultures (Liu et al., 2017). Indeed, both CR and organoids technologies started with normal stem-like cell types in early 2010. We also highlighted importance of pathological evaluation before CR culture. Technically there are three impossible tasks: First, there is no existing technology available for select tumor or normal cells or other cell types in fresh tissue samples without formalin-fixation and H&E staining; Second, there is no existing technique available for allowing to proportionally propagate all cell types in the specimens at the same time under same culture conditions; Third, there is no existing technique available for selection of normal cell cultures or tumor cell cultures from the fresh specimens. In a study by Gao et al., samples from 22 normal lungs and 48 primary non-small cell lung cancer patients were expanded using the CR technique (Gao et al., 2017). They found that normal and tumorous lung epithelial cells could be cultured. The majority of the cells produced by co-culturing non-malignant lung epithelial and tumor cells were non-malignant. Only two of the 22 mutations identified in the original tumors were detected with a lower frequency in the CRC cultures (Gao et al., 2017). Sette and colleagues found that cultures derived from NSCLC biopsies quickly lost genetic mutations specific to the patient or tumor antigens (Sette et al., 2018a). Hynds et al. obtained primary epithelial cell cultures that could be established from 10 of 12 lung adenocarcinoma samples. In CR culture, tumor-derived cells displayed characteristics that were identical to those of normal human airway epithelial cells. Cancer mutation-bearing cells were found in only 1 out of 10 early passage patient cultures (Hynds et al., 2018). Thus, success rates of CR lung cells from patients have been variable in literature, indicating that CR conditions preferably support the growth of normal airway epithelial cells (Gao et al., 2017; Hynds et al., 2018; Sette et al., 2018a). While these studies failed to describe percentage of cancer cells in their specimens as the above studies (Crystal et al., 2014; Piotrowska et al., 2015), side-by-side comparison, optimization of culture conditions and prior selection of cancer cells from tumor specimens are critical to clarify the applicability of CR conditions in lung cancer studies.

### 4.2 Selection of cell types during initial and continued CR cultures

Selective culture of tumor cells is hindered due to the absence of reliable assays and biomarkers to differentiate between normal and tumor epithelial cells in CR cultures. DNA methylation patterns are one of the promising assays enabling the differentiation of normal from tumor epithelial cells. Cellular development and differentiation are mainly regulated by DNA methylation patterns (Callinan et al., 2006). One of the main epigenetic characteristics of human malignancies, including lung cancer, is aberrant DNA methylation. (Belinsky, 2004; Dietrich et al., 2012). According to prior studies, lung cancer tissues had higher levels of promoter methylation for PTGER4 and short stature homeobox gene two (SHOX2) when compared to matched, normal tissues (Ilse et al., 2013, 2014). In this regard, in an attempt to identify malignant epithelial cells in CR cultures, Wu and colleagues developed a PCR-based DNA methylation assay that investigates the promoters of the SHOX2 and PTGER4 genes (Wu et al., 2020c). Firstly, they discovered that 28 out of 31 (90%) malignant lung samples exhibited elevated methylation of the SHOX2 and/or PTGER4 promoters when compared to the adjacent normal samples. A further observation made in 13 out of 25 (52%) CR cultures was increased methylation of the SHOX2 or PTGER4 promoter regions. Growth in soft agar cultures, a sign of malignant transformation, and EGFR mutation analysis verified the existence of malignant cells. These findings show that malignant lung epithelial cells in CR cultures can be identified by measuring the levels of promoter methylation of SHOX2 and PTGER4 (Wu et al., 2020c). Utilizing the CR technique, Blazek et al. assessed the primary culture radiosensitivity parameters for both large single and multiple radiation fractions used in Simulated Stereotactic Body Radiation Therapy (SBRT) (Blazek et al., 2020). two of four freshly resected tumor samples were collected and established. 0.6 Gy/min of radiation was applied using a^137^Cs irradiator. Within four weeks of plating, plates were stained and scored, and the linear-quadratic model was fitted to the clonogenic survivals. One patient's primary cells (L343 E/M) were intermediate in the range of radiosensitivities for cultivated NSCLC lines. Despite using the measured a/b value instead of assuming a/b = 10, the effective survivals for several conventional and SBRT fractions were different from those predicted by the time-independent biologically effect dose. Based on Blazek and colleagues' study the CR technique might be an ideal tool for genomic personalization of radiation therapy and can be used to determine patient radiosensitivity parameters for NSCLC patients (Blazek et al., 2020). A study was conducted by Talwelkar et al. to tailor precision medication in lung cancer patients (Talwelkar et al., 2021). Talwelkar and associates established NSCLC patient-derived cultures using the CR technique. Targeted next-generation sequencing was carried out on 11 primary cultures, matched tumors, and adjacent normal lung tissues to identify 578 cancer-related genes. 9 of the 11 cultures lacked the oncogenic mutations detected in the parental tumor. Interestingly, the two cultures that demonstrated consistent alterations with their respective tissue were derived from various parts of the same tumor. As we described above, there is no existing technology available for selecting tumor or normal cells or other cell types in fresh tissue samples without formalin-fixation and H&E staining. Thus, it is extremely important to know how much tumor tissue is used for the generation of initial CR cultures. Again, development of selection techniques in initial and continued CR cultures would be critical for generation of stable CR cell lines. As we discussed above, subsequently passaging off the feeder layer for further expansion may be critical since those resulting CR lung cancer cells retained all key mutations and resistant phenotypes that were identified in the corresponding patient tumors (Crystal et al., 2014; Piotrowska et al., 2015). Although the below case is from prostate cancer CR cultures, it is possibly applicable to several other cancer types. Various culture conditions were tested to identify factors that allowed the proliferation of tumor cells, but did not support expansion of normal cells. We found that culturing CRs in DMEM without serum for 3 days led to differentiation of normal prostate CR cells, while supporting a “fibroblast”-like or mesenchymal morphologic phenotype of tumor CR cells (GUMC-30). Interestingly, CR conditions reversed “fibroblast”- or mesenchymal-like cells back to epithelial morphology (Timofeeva et al., 2017). This suggests that differential selection is possible for outgrowth of either normal or tumor CR cells after initial CR co-cultures. Thus, normal CR can be used for studies of cell biology, proliferation and differentiation, tissue repair, and cancer initiation, while tumor CR cells can be used for studies of cancer biology, cancer initiation and progression, responses and resistance. As we discussed, CR have been used to generate valuable living cell banks from upper aerodigestive tumors with tumorigenicity and similar responses to treatments as patients.

## 5 Regenerative medicine using CRCs

Regenerative medicine involves employing biotechnological methods to repair, replace, or regenerate cells, tissues, or organs that have been damaged or affected by diseases. This field includes various novel methods, such as stem cell therapy, tissue engineering, immunomodulatory therapy, gene therapy, and other biological interventions (Parekh et al., 2020). Recently, considerable attention has been directed towards investigating the potential of various stem cell types, including adult stem cells, iPSCs, and embryonic stem cells in regenerative medicine (Wagers, 2012). However, there exists some challenges and difficulties in effectively inducing the functional differentiation of stem cells. Given the potential of CR technology which is able to generate cells from very few donor tissues and can rapidly expand them in vitro in large quantities and subsequently differentiate into origin cells upon the removal of CR conditions, CR technology shows great promise for various applications in tissue repair and regenerative medicine. Butler and colleagues utilized the CR technique and uncovered its potency in the rapid proliferation of functional human airway basal cells (Butler et al., 2016). These cells demonstrated pluripotent differentiation potential in vitro conditions and successfully populated tracheal scaffolds in a transplantation xenograft model, indicating their feasibility for tracheal reconstruction (Butler et al., 2016). In another study, LaRanger and colleagues discovered that within 12 days of being implanted into the decellularized mouse lung, CR bronchial epithelial cells demonstrated differentiation into both upper airway bronchial epithelium and lower airway alveolar architecture (LaRanger et al., 2018). Consistently, Hamilton et al. employed a rabbit model of revascularization, incorporating a combination of airway epithelial CR cells and a fibroblast-containing graft, alongside decellularized tracheal stents and implanted structures designed for revascularization. Throughout the scaffold, they observed the presence of keratin-positive cells (Hamilton et al., 2019). Their study indicated that CR cells have the potential to enhance host epithelium repair and/or directly facilitate mucosal regeneration, offering promising applications in regenerative medicine. Additionally, through CR method, primary airway epithelial cells can be established from cryopreserved biopsy specimens (Gowers et al., 2018). This capability enables the transfer of samples between clinical facilities and specialist laboratories, potentially revolutionizing biobanking repositories (Palechor-Ceron et al., 2019). Moreover, various research groups have employed CRISPR-Cas9 gene editing technology with CR cells to explore molecular mechanisms. For example, applying CRISPR-Cas9 genetic editing to CR cells uncovered the pro-inflammatory effects of MUC18 in airway epithelial cells and elucidated the role of the NLRP1 Inflammasome in UVB sensing in human primary keratinocytes (Chu et al., 2015; Fenini et al., 2018). These genome editing endeavors are likely to catalyze further research and potential medical applications employing CR technology.

## 6 Challenges and future prospects

Despite the promising advancements of CR in cancer research, numerous challenges associated with this technique still need to be addressed. First, in relation to the culture system, CRCs can become contaminated with feeder cells, potentially interfering with subsequent assays (Palechor-Ceron et al., 2013). To address this limitation, Liu et al. discovered that direct physical contact between feeder cells and epithelial cells is not necessary for the induction of CR and immortalization. Therefore, contamination may be prevented by using a simplified culture system that replaces feeder cells with a medium conditioned by irradiated feeder cells (Palechor-Ceron et al., 2013).

Second, Maintaining the accuracy of tumor-derived genetic alterations and tumor phenotypes continues to be a significant challenge, as highlighted in reports for specific cancer types (Sette et al., 2018b; Yu et al., 2017b). In nasopharyngeal carcinoma, Yu et al. reported that only 40% of the tumor-derived CRCs were able to preserve some of the mutant genes identified in their original parental samples. (Yu et al., 2017b). In lung cancer, genetic mutations in tumor-derived CRCs were found to be absent, with all samples showing a complete loss of these mutations by the fourth passage (Sette et al., 2018b). CRCs that lack tumor-specific alterations and phenotypes may potentially be classified as normal epithelial cells or could result from the excessive proliferation of non-malignant cells. In the future, modifications to the existing CR culture system may be implemented to enhance the efficiency of deriving malignant CRCs and to ensure the accurate representation of genetic aberrations and tumor phenotypes.

Third, The CR method is limited by its exclusion of critical stromal components, such as matrix elements, vascular immune cells, and endothelial cells. This shortcoming impedes a thorough understanding of how stromal cells affect tumor cell growth and how tumor cells react to drug treatments (Palechor-Ceron et al., 2013). Despite these limitations, CR technology presents promising potential for research in the respiratory tract. No single model is ideal for all biomedical research; therefore, researchers must select the model that best addresses their specific research question. Often, scientists employ a combination of technologies across various levels—from molecules to cells, organs, and populations—to achieve their research objectives.

## 7 Conclusions

In a brief period of development and implementation, CR has emerged as a potent tool for establishing primary epithelial cell cultures indefinitely. The advent of CR technology holds promising potential for investigating respiratory diseases and normal tissues. A significant feature of CR technology is that the withdrawal of ROCK inhibitors and feeder cells enables CRCs to undergo normal differentiation. Additionally, the CR system facilitates rapid and efficient generation of cell cultures from both normal and malignant tissues, preserving the developmental characteristics of the original tissue and maintaining tumor heterogeneity. Furthermore, CR technology enables rapid generation of cultures from small biopsy specimens, brushings, swaps, cryopreserved tissues, as well as from organoid tissues and xenografts. This capability supports the establishment of cell-derived xenograft tumors and organoids, positioning CR technology as a potentially ideal in vitro model for respiratory tract research. It holds promise for advancing personalized medicine, regenerative medicine, and accelerating drug discovery efforts. Moreover, in the future, CR technology could potentially create a living biobank for various respiratory tract normal and malignant tissues.

## CRediT authorship contribution statement

**Danyal Daneshdoust:** Writing – review & editing, Writing – original draft, Resources, Investigation, Conceptualization. **Kai He:** Writing – review & editing, Resources. **Qi-En Wang:** Writing – review & editing, Resources. **Jenny Li:** Writing – review & editing, Resources, Investigation, Conceptualization. **Xuefeng Liu:** Writing – review & editing, Supervision, Resources, Methodology, Conceptualization.

## Declaration of competing interest

Several patents for CR technology have been awarded to Georgetown University by the US Patent Office. The license for this technology has been given to a Maryland-based start-up company for commercialization. The inventor, X.L., and Georgetown University receive potential royalties and payments from the company. CR media and CR cells have been distributed by Propagenix (acquired by StemCell Technologies), Fisher Scientific, ATCC, etc. Other authors declare that they have no known competing financial interests or personal relationships that could have appeared to influence the work reported in this paper.
